# Changes in subcutaneous adipose tissue microRNA expression in response to exercise training in African women with obesity

**DOI:** 10.1038/s41598-022-23290-x

**Published:** 2022-11-01

**Authors:** Carmen Pheiffer, Stephanie Dias, Amy E. Mendham, Babalwa Jack, Tarryn Willmer, Nasr Eshibona, Hocine Bendou, Ashley Pretorius, Julia H. Goedecke

**Affiliations:** 1 Biomedical Research and Innovation Platform, South African Medical Research Council, Tygerberg, 7505 South Africa; 2 Centre for Cardio-Metabolic Research in Africa (CARMA), Division of Medical Physiology, Faculty of Medicine and Health Sciences, Stellenbosch University, Tygerberg, 7505 South Africa; 3 Department of Obstetrics and Gynaecology, Faculty of Health Sciences, University of Pretoria, Pretoria, 0001 South Africa; 4 South African Medical Research Council/WITS Developmental Pathways for Health Research Unit (DPHRU), Department of Paediatrics, School of Clinical Medicine, Faculty of Health Sciences, University of the Witwatersrand, Johannesburg, 2050 South Africa; 5 Division of Physiological Sciences, Department of Human Biology, Health Through Physical Activity, Lifestyle and Sport Research Centre (HPALS), FIMS International Collaborating Centre of Sports Medicine, Faculty of Health Sciences, University of Cape Town, Observatory, 7925 South Africa; 6 South African Medical Research Council Bioinformatics Unit, South African National Bioinformatics Institute, University of the Western Cape, Bellville, 7535 South Africa; 7 Diagnostic Aptamer Technologies, Aminotek, Cape Town, 8000 South Africa

**Keywords:** Metabolic disorders, miRNAs

## Abstract

The mechanisms that underlie exercise-induced adaptations in adipose tissue have not been elucidated, yet, accumulating studies suggest an important role for microRNAs (miRNAs). This study aimed to investigate miRNA expression in gluteal subcutaneous adipose tissue (GSAT) in response to a 12-week exercise intervention in South African women with obesity, and to assess depot-specific differences in miRNA expression in GSAT and abdominal subcutaneous adipose tissue (ASAT). In addition, the association between exercise-induced changes in miRNA expression and metabolic risk was evaluated. Women underwent 12-weeks of supervised aerobic and resistance training (n = 19) or maintained their regular physical activity during this period (n = 12). Exercise-induced miRNAs were identified in GSAT using Illumina sequencing, followed by analysis of differentially expressed miRNAs in GSAT and ASAT using quantitative real-time PCR. Associations between the changes (pre- and post-exercise training) in miRNA expression and metabolic parameters were evaluated using Spearman’s correlation tests. Exercise training significantly increased the expression of miR-155-5p (1.5-fold, p = 0.045), miR-329-3p (2.1-fold, p < 0.001) and miR-377-3p (1.7-fold, p = 0.013) in GSAT, but not in ASAT. In addition, a novel miRNA, MYN0617, was identified in GSAT, with low expression in ASAT. The exercise-induced differences in miRNA expression were correlated with each other and associated with changes in high-density lipoprotein concentrations. Exercise training induced adipose-depot specific miRNA expression within subcutaneous adipose tissue depots from South African women with obesity. The significance of the association between exercise-induced miRNAs and metabolic risk warrants further investigation.

## Introduction

Obesity is recognized as a worldwide epidemic that increases the risk for metabolic diseases such as insulin resistance and type 2 diabetes^1^. Adipose tissue location is a key determinant of metabolic risk^2^. Excessive fat accumulation within adipose depots in the abdominal or android region is associated with greater metabolic risk, while lower-body fat in the gynoid region is thought to be protective against metabolic disease^3,4^. The mechanisms that underlie the association between adipose tissue depot and metabolic risk are not known, although studies have suggested that epigenetic and transcriptome profiles may account for the functional differences and consequences for disease^2,5,6^.

MicroRNAs (miRNAs) have emerged as powerful epigenetic regulators of a variety of developmental processes and disease^7,8^. MiRNAs are single-stranded, non-coding RNA molecules approximately 22 nucleotides in length that bind to the 3′ untranslated region (UTR) of messenger RNA (mRNA) inducing degradation or translational repression of the mRNA transcript^9^. Accumulating literature reports that miRNAs regulate gene expression in response to environmental and nutritional cues to orchestrate cellular responses in obesity and insulin resistance^10^. In adipose tissue, miRNAs regulate adipocyte differentiation and tissue expansion, with over 40 miRNAs correlated with obesity and type 2 diabetes^10,11^. Adipose tissue miRNAs have also been implicated in the control of metabolic and inflammatory processes^12^, and have been shown to vary between adipose depots^5^. In recent years, a growing body of evidence have reported that miRNAs are key players in adaptive responses to exercise in patients with obesity and obesity-related disorders^13^.

Exercise training is an important non-pharmacological strategy that prevents obesity and metabolic diseases by stimulating lipid catabolism in adipose tissue leading to decreased adipose mass and improving whole-body metabolic health^14,15^. Exercise induces lipolysis, a process whereby triacylglycerols, an energy reservoir in adipose tissue, are hydrolyzed to free fatty acids (FAs), which are released into circulation providing fuel to skeletal muscle. The beneficial effects of exercise may be mediated by epigenetic mechanisms in adipose tissue^14^ and may be adipose depot specific^5^.

This study aimed to investigate miRNA expression in gluteal subcutaneous adipose tissue (GSAT) in response to a 12-week exercise intervention in South African women with obesity, and to assess depot-specific differences in miRNA expression in GSAT and abdominal subcutaneous adipose tissue (ASAT). In addition, we evaluated the association between exercise-induced changes in miRNA expression and metabolic risk.

## Results

### Participant characteristics

The characteristics of participants in this study have been described in detail previously^16,17^ and are summarized in Table 1. All participants were obese (body mass index (BMI) > 30 kg/m^2^), insulin resistant, and between 21 and 28 years of age. The 12-week exercise intervention resulted in a significant increase in peak oxygen consumption (VO_2_peak, p = 0.007), an indicator of cardiorespiratory fitness, as well as insulin sensitivity (S_I_, p = 0.042) and circulating triglycerides levels (p = 0.002). In contrast, waist circumference (p = 0.001), and gynoid fat mass (p = 0.010) decreased in response to exercise training. Waist circumference of the control group increased during the 12-week experimental period (p = 0.018). Energy intake, macronutrient distribution, physical activity and alcohol consumption remained unchanged in the exercise and control groups (Supplementary Table S5).

**Table 1:** Participant’s characteristics before and after the 12-week intervention.

Variable	Control (n = 12)		Exercise (n = 19)		Group	Time	Interaction
	Pre	Post	Pre	Post	P value	P value	P value
Age (years)	24 (22;28)	–	22 (21;24)	–	–	–	–
VO_2_peak (ml/min)	2085 (313)	2021 (212)^a^	2090 (210)^b^	2289 (232)^a,b^	0.961	0.406	0.007
VO_2_peak (ml/min/kg)	23.5 (3.1)	22.6 (2.7)^c^	24.8 (2.4)^b^	27.5 (3.4)^b,c^	0.211	0.286	0.001
**Body fat distribution**							
Weight (kg)	85.6 (79.1;94.5)	87.2 (81.0;95.5)	83.0 (78.7;91.6)	82.6 (76.1;92.0)	0.306	0.047	0.006
Body mass index (kg/m^2^)	33.0 (31.1;36.5)	33.3 (31.6;36.5)	34.9 (32.8;36.4)	34.7 (30.4;37.0)	0.399	0.047	0.006
Waist circumference (cm)	103.5 (8.9)^a^	106.7 (8.5)^a^	103.9 (7.4)^b^	100.7 (8.8)^b^	0.886	0.003	< 0.001
Waist-to-hip ratio	0.87 (0.1)	0.90 (0.1)	0.91 (0.1)	0.89 (0.1)	0.150	0.034	0.005
Fat mass (%)	49.1 (47.0;51.9)	49.6 (47.3;52.3)	50.5 (48.5;52.6)	50.2 (48.3;52.8)	0.305	0.695	0.579
Android fat mass (% fat mass)	8.3 (1.4)	8.1 (1.6)	8.3 (1.0)	8.2 (1.0)	0.901	0.098	0.781
Gynoid fat mass (% fat mass)	19.4 (2.6)	19.5 (2.6)	18.4 (1.7)^a^	18.2 (1.6)^a^	0.217	0.197	0.003
VAT (cm^3^)	981.6 (472.5)	1014.2 (427.5)	931.4 (326.7)	916.3 (353.4)	0.662	0.276	0.244
SAT (cm^3^)	5630.2 (1873.5)	5662.2 (1968.2)	5603.9 (945.2)	5567.0 (1169.3)	0.915	0.044	0.065
**Metabolic parameters**							
Fasting glucose (mmol/L)	4.9 (0.6)	5.1 (0.8)	5.5 (0.8)	5.1 (1.0)	0.048	0.533	0.148
Fasting insulin (pmol/L)	13.4 (11.2;18.7)	14.3 (11.4;20.0)	14.8 (6.4;19.1)	12.5 (10.5;17.1)	0.428	0.853	0.926
HOMA-IR	2.9 (2.4;4.3)	3.4 (2.8;4.3)	3.6 (1.6;5.2)	3.2 (2.1;4.7)	0.856	0.663	0.543
S_I_ (× 10^–4^ min^−1^/(uU/ml)	2.0 (1.3;3.2)	1.8 (1.6;2.6)	2.0 (1.2;2.8)^a^	2.2 (1.5;3.7)^a^	0.204	0.680	0.049
Leptin (ng/ml)	70.1 (17.5)	73.4 (21.6)	67.1 (24.9)	65.4 (22.6)	0.713	0.459	0.389
C-reactive protein (µg/ml)	2.5 (2.0;8.7)	4.0 (2.5;7.8)	6.1 (2.2;13.8)	4.9 (2.8; 9.1)	0.312	0.159	0.263
TNFα (pg/ml)	7.4 (6.1;9.1)	7.6 (5.6;12.5)	5.2 (3.3;8.4)	4.6 (4.0;9.3)	0.285	0.201	0.407
Triglycerides (mmol/L)	0.8 (0.6;1.1)	0.7 (0.5;0.9)	0.7 (0.6;0.8)^b^	0.9 (0.7;1.0)^b^	0.092	0.187	0.001
LDL (mmol/L) HDL (mmol/L) triglycerides (mmol/L)	2.0 (1.6;2.7)	1.7 (1.4–2.9)	2.7 (2.0;3.1)	2.7 (1.9;3.5)	0.301	0.388	0.442
HDL (mmol/L)	0.95 (0.21)	0.97 (0.21)	1.01 (0.23)	1.05 (0.18)	0.442	0.761	0.825
Total cholesterol (mmol/L)	3.9 (1.3)	3.5 (1.2)	4.0 (0.8)	4.3 (0.9)	0.703	0.240	0.122

Data are presented as the mean ± SD for normally distributed variables or as the median and interquartile range for skewed variables.

HDL, high density lipoprotein; HOMA-IR, homeostatic model of insulin resistance; LDL, low density lipoprotein; S_I_, insulin sensitivity; TNFα, tumor necrosis factor alpha; VO_2_peak, peak rate of oxygen consumption.

^a^p < 0.05; ^b^p < 0.01; ^c^p < 0.001.

### Exercise-induced microRNAs identified using Illumina sequencing

The effect of exercise training on miRNA expression in GSAT was investigated using Illumina sequencing. GSAT was selected for sequencing based on previous findings which showed that GSAT is more metabolically active than ASAT in our population^2^. MiRNAs isolated from GSAT pre- and post-experimental period were sequenced in the exercise group (n = 8) and controls (n = 4) to identify exercise-induced miRNAs. On average 10,844,112 clean reads and 10,733,160 adapter-trimmed reads (length ≥ 15 nucleotides) were identified, of which, 8,412,296 aligned to known human pre-miRNA in miRBase21 (http://mirbase.org). MiRNAs identified by sequencing are listed in the Supplementary Table S1. MiRNAs that showed a significant difference in response to the exercise training intervention were selected for subsequent qRT-PCR (Table 2).

**Table 2:** MiRNA fold-regulation in GSAT pre- and post-exercise training.

MiRNA	Fold regulation	P value
**Increased**		
MiR-155-5p	↑ 1.7	0.035
MiR-329-3p	↑ 1.2	0.045
MiR-377-3p	↑ 1.2	0.011
MYN0617^a^	↑ 1.1	0.024
**Decreased**		
MiR-676-3p	↓ 1.1	0.002
MiR-1306-5p	↓ 1.1	0.030

MiRNA expression was quantified as transcripts per million of total aligned miRNA Illumina reads.

Fold regulation represents miRNA expression^post-exercise^/_pre-exercise_.

^a^hsa-miR-novel-chr3_31164.

### Effect of exercise intervention on miRNA expression

**Figure 1:**
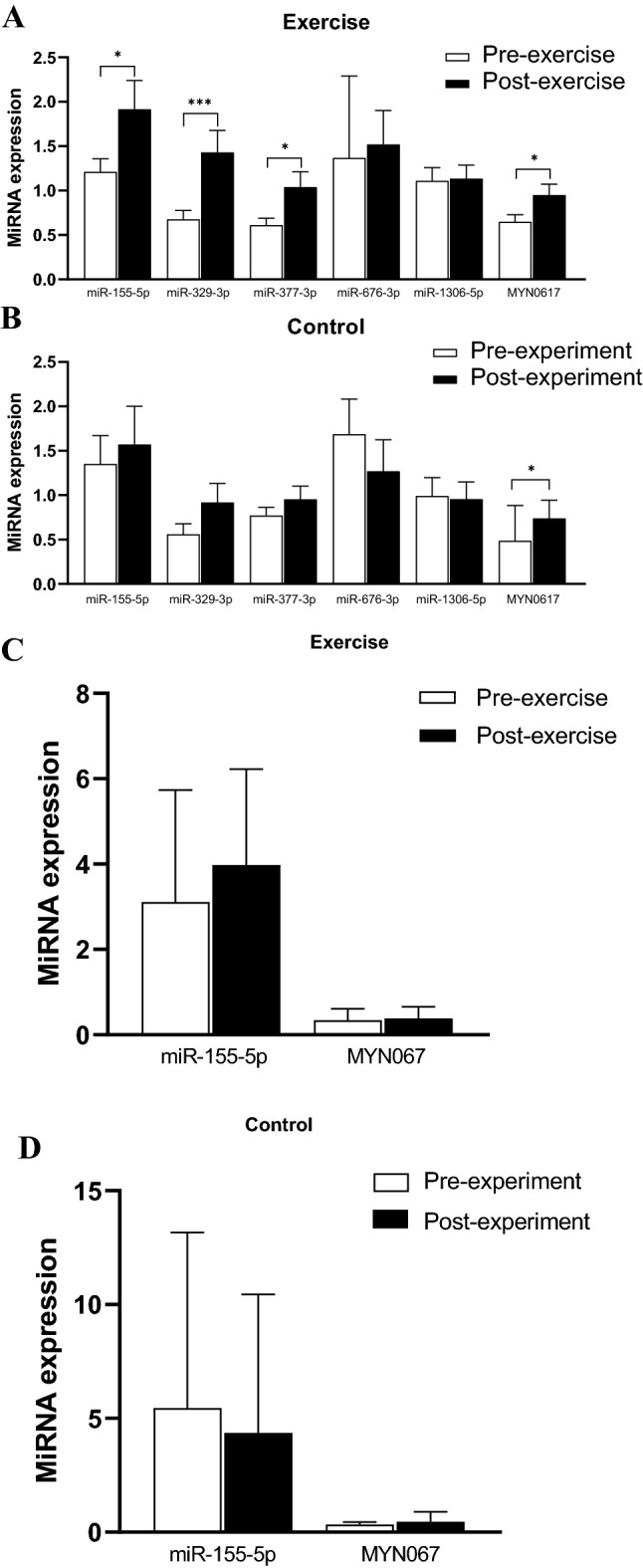
MiRNA expression in GSAT in the exercise training (**A**) and control (**B**) groups, and in ASAT in the exercise training (**C**) and control (**D**) groups. MiRNA expression was quantified using TaqMan qRT-PCR. Results are expressed as the mean ± SD (exercise group n = 19; controls n = 12). *p < 0.05, ***p < 0.001.

TaqMan quantitative real-time PCR (qRT-PCR) was conducted to confirm the differential expression of miRNAs observed by sequencing. MiR-155-5p (↑ 1.5-fold, p = 0.045), miR-329-3p (↑ 2.1-fold, p < 0.001), miR-377-3p (↑ 1.7-fold, p = 0.013) and MYN0617 (↑ 1.5-fold, p = 0.012) showed increased expression in GSAT in response to exercise training, thus confirming sequencing results (Fig. 1A). The expression of miR-155-5p, miR-329-3p, miR-377-3p, miR-676-3p and miR-1306-5p did not change in the controls (p < 0.05), however, the expression of MYN067 was increased 1.5-fold (p = 0.042) in the control group over the 12-week period (Fig. 1B). In ASAT, the expression of miR-155-5p increased 1.3-fold after exercise training, however, the difference was not statistically significant (p < 0.05) (Fig. 1C). Low expression of MYN067 was observed in ASAT. MiR-329-3p and miR-377-3p were not detected in ASAT. No difference in the expression of miR-155-5p was observed in the control group (Fig. 1D). In a paired analysis of the combined sample (exercise and control groups) at baseline the expression of miR-155-5p was approximately 3.6-fold (p < 0.001) higher in ASAT compared to GSAT (Supplementary Figure S1).

### Correlations between exercise-induced miRNAs and associations with metabolic risk

**Figure 2:**
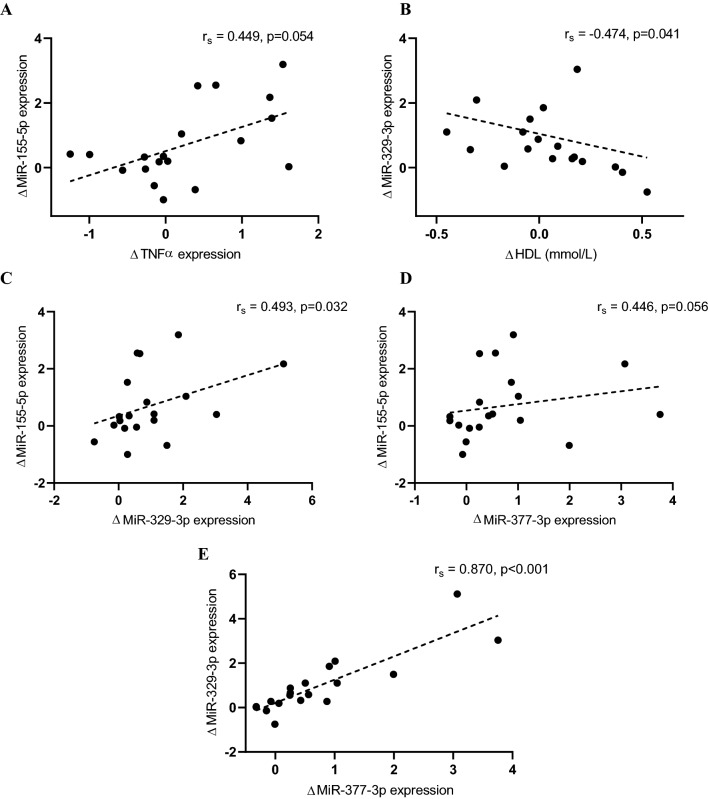
Spearman’s correlation analysis of the post-training change from baseline (Δ) in miR-155-5p expression and changes in TNFα gene expression in GSAT (**A**), miR-329-3p expression in GSAT and high-density lipoprotein (HDL) levels (**B**), miR-155-5p and miR-329-3p expression in GSAT (**C**), miR-155-5p and miR-377-3p expression in GSAT (**D**) and miR-329-3p and miR-377-3p expression in GSAT (**E**). Each point represents a participant. Linear regression lines used for descriptive purposes only.

We next assessed associations between the post-training change from baseline (Δ) in miRNA expression and metabolic risk (Fig. 2). Exercise-induced changes in miR-155-5p levels in GSAT were correlated with TNFα gene expression in GSAT, although the association was not statistically significant (r_s_ = 0.449, p = 0.054). A negative correlation between miR-329-3p (r_s_ = − 0.474, p = 0.041) levels in GSAT and high-density lipoprotein (HDL) concentrations were observed. In addition, changes in miRNA levels were positively correlated with each other. MiR-155-5p levels were correlated with miR-329-3p (r_s_ = 0.493, p = 0.032) and miR-377-3p (r_s_ = 0.446, p = 0.056), while miR-329-3p and miR-377-3p were correlated with each other (r_s_ = 0.870, p < 0.001).

### Increased miR-155-5p expression during conditions of obesity, inflammation and lipolysis

**Figure 3:**
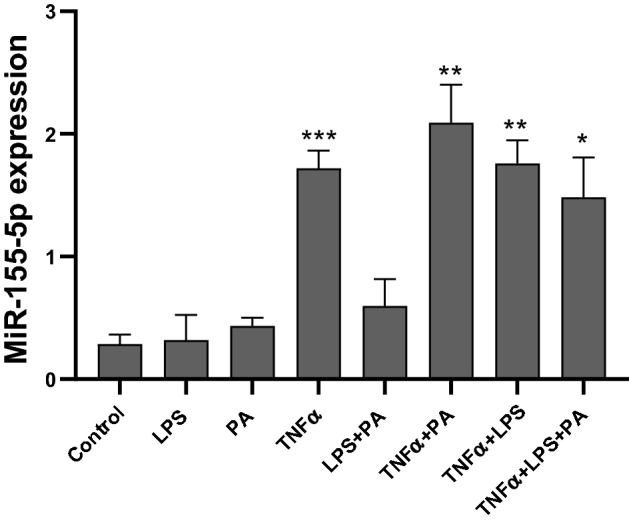
MiR-155-5p expression in 3T3-L1 adipocytes. Differentiated 3T3-L1 adipocytes were treated with 100 ng/ml LPS, 750 µM PA and 10 ng/ml TNFα or a combination of these compounds. MiRNA expression was quantified using qRT-PCR. Results are expressed as the mean ± SD of triplicate experiments. Significance is depicted vs. control. *p < 0.05, **p < 0.01, ***p < 0.001. Abbreviations: lipopolysaccharide, LPS; palmitic acid, PA; tumor necrosis factor alpha, TNFα.

We next assessed the expression of miRNAs in a cell model of obesity, inflammation and lipolysis commonly used to decipher the molecular mechanisms that underlie human obesity^18^. 3T3-L1 adipocytes were exposed to lipopolysaccharide (LPS), palmitic acid (PA) and tumor necrosis factor alpha (TNFα) to stimulate inflammation and dyslipidemia. The expression of miR-155-5p was increased in lipolytic and TNFα-, but not LPS- and PA- exposed adipocytes (Fig. 3). MiR-329-3p, miR-377-3p and MYN0617 were not detected in 3T3-L1 adipocytes.

### Bioinformatic analysis

MiR-155-5p, miR-329-3p, miR-377-3p and MYN067 were associated with various pathways associated with lipid metabolism and insulin signaling (Supplementary Table S3 and Table S4).

## Discussion

A growing body of evidence demonstrates that exercise training improves obesity and metabolic health by modulating miRNA expression in adipose tissue^13^. The current study provides novel evidence to show that exercise training induces miRNA expression differences in adipose tissue from South African women with obesity and insulin resistance, which occurs in an adipose-depot specific manner. Furthermore, we show that the exercise-induced miRNA differences correlated with changes in measures of metabolic risk in response to exercise.

Exercise training is an important non-pharmacological strategy to prevent obesity and metabolic diseases^19,20^. While both aerobic and resistance training improve health, the combination of aerobic and strength training has been recommended as an effective strategy for weight loss and to promote metabolic health^21,22^. Therefore, as anticipated, combined exercise training increased cardiorespiratory fitness and insulin sensitivity, and decreased gynoid fat mass and waist circumference. Cardiorespiratory fitness^23^ and insulin sensitivity^24^ are inversely associated with metabolic risk, while waist circumference is a measure of central obesity and has been identified as a modifiable risk factor for insulin resistance and cardiometabolic disease^25^. These findings show that 12-weeks of an exercise training program with combined aerobic and strength training reduced metabolic risk in South African women with obesity and insulin resistance and demonstrates the benefits of exercise in our population.

High levels of circulating triglycerides are associated with obesity and metabolic risk^26^, therefore, the finding of increased triglyceride levels in response to exercise training may seem counterintuitive. Previous studies have suggested that exercise training prevents obesity and metabolic diseases by stimulating lipolysis in adipose tissue and releasing FAs into circulation to be utilized by skeletal muscle as fuel during exercise^14,15^. Our findings of increased expression of the exercise-induced miR-155-5p in GSAT as well as in an adipocyte cell model of lipolysis and inflammation, suggests that the exercise intervention may decrease gynoid fat mass and waist circumference by inducing miR-155-5p expression and stimulating lipolysis, thereby increasing circulating triglycerides. A previous study using the same study participants as us showed that the 12-week exercise intervention reduced gynoid fat mass through increased fat oxidation^27^. Rydén and Arner investigated lipolysis regulation in adipose tissues from one thousand and sixty-six men and women and reported a significant correlation between lipolysis and circulating triglyceride levels^28^, while Vechetti et al. reported increased miR-1 expression and lipolysis in white adipose tissue in response to exercise training^29^.

MiRNA profiling revealed that 12-weeks of exercise training significantly increased miR-155-5p, miR-329-3p and miR-377-3p in GSAT, but not in ASAT. Moreover, miR-329-3p and miR-377-3p were not detected in ASAT. A novel miRNA, MYN067, which was increased in GSAT after the exercise intervention, was shown to be associated with biological pathways related to insulin and glucose metabolism. MYN067 was increased in the control group as well, illustrating that the induction of this miRNA over the study period occurred independent of the exercise intervention and could be due to other environmental or dietary factors. Our inability to detect significant differences in ASAT could be due to the smaller sample size available for this depot or due to inherent differences between GSAT and ASAT, as previous studies by Nono Nankam et al. demonstrated whole-genome transcriptomic^30^ and inflammatory gene expression^31^ differences in these tissues from the same study population. The exercise-induced change in expression of miR-329-3p in GSAT was negatively correlated with changes in HDL concentrations. To the best of our knowledge, these adipose-depot dependent miRNA expression differences and associations with metabolic risk have not been previously described and warrants further work to determine the significance of these associations.

MiR-155-5p knockdown experiments in mice and cell models provide clues about its function, albeit with inconsistent results. Velázquez et al. demonstrated that miR-155 deletion in high‐fat diet (HFD) fed male mice exacerbated adipose tissue fibrosis^32^. Another study where miR-155 was knocked down in HFD fed female and male mice showed that miR-155 deletion prevents fat accumulation in female, and to a lesser extent, in male mice^33^. However, in contrast, another study found that knockdown of miR-155 was associated with increased adipogenesis and greater expression of three key adipogenic transcription factors, CCAAT/enhancer-binding protein alpha (C/EBPα), C/EBP beta (C/EBPβ), and peroxisome proliferator-activated receptor gamma (PPARγ)^34^. A mouse knockdown model provided evidence that miR-155 is a positive regulator of insulin sensitivity with potential applications for diabetes treatment^35^. This study showed that overexpression of miR-155 in female and male mice improved glucose tolerance and insulin sensitivity, while conversely, miR-155 deficiency caused hyperglycemia, impaired glucose tolerance and insulin resistance. These effects were suggested to be mediated through increased Protein kinase B (AKT) and Insulin receptor substrate 1 (IRS1) phosphorylation in liver, adipose tissue and skeletal muscle cells^35^. In vitro studies in 3T3-L1 adipocytes demonstrated that increased expression of miR-155 was associated with decreased adipogenesis^36^, while miR-155 knockdown in preadipocytes increases adipogenesis^33^. Given the discrepant results of these studies, it is evident that more studies are needed to elucidate the role of miR-155 in obesity development.

**Figure 4:**
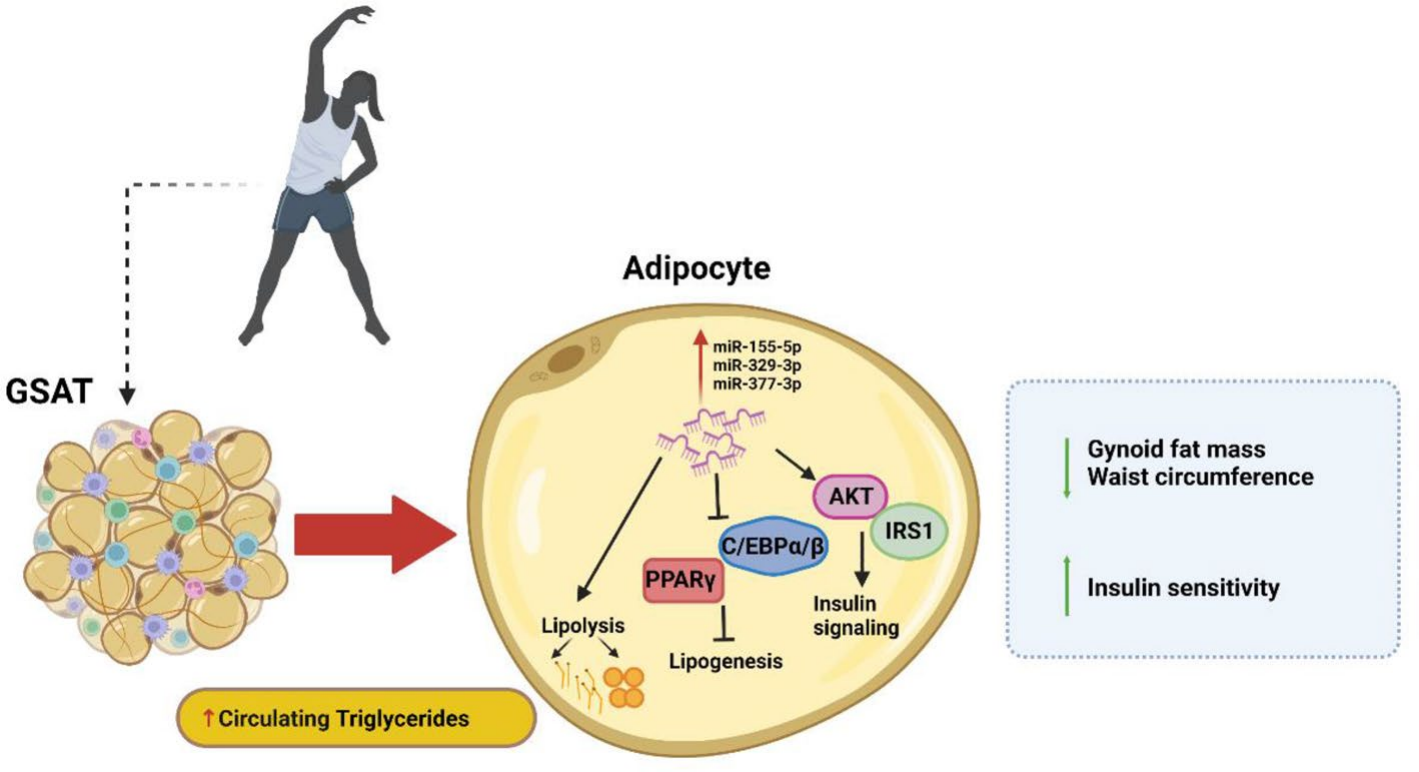
Proposed model of study findings showing how the exercise intervention can lead to reduced waist circumference and gynoid fat mass and improve whole-body insulin sensitivity. Exercise induces the expression of miR-155-5p, miR-329-3p and miR-377-3p in GSAT, stimulating lipolysis and increasing circulating triglycerides. Inhibition of C/EBPα/β and PPARγ leads to decreased lipogenesis, with concomitant stimulation of AKT and IRS1, and insulin signaling. Abbreviations: AKT, Protein kinase B; C/EBPα/β, CCAAT/enhancer-binding protein alpha/beta; GSAT, Gluteal subcutaneous adipose tissue; IRS1, Insulin receptor substrate 1. Diagram created in Bio-Render (https://biorender.com/).

Our findings are in contrast to a previous study that reported no difference in plasma miR-155-5p expression in response to marathon running in male athletes^37^. MiR-155-5p is a pro-inflammatory miRNA that is increased during macrophage differentiation to a M1 or inflammatory phenotype^38^. A study by Bao et al. reported that an acute exercise session consisting of 30 min of moderate intensity on a treadmill induced the expression of inflammatory circulating miRNAs in obese individuals^39^, although miR-155 levels were not measured in this study. Our in vitro findings showed that miR-155-5p is increased in adipocytes exposed to TNFα, but not in LPA or PA treated adipocytes, supporting its pro-inflammatory properties. Another exercise-induced miRNA, miR-329-3p, has been reported to possess insulin sensitizing effects^40,41^, while miRNA, miR-377, has an important regulatory role in adipogenesis and triglyceride metabolism^42,43^. Bioinformatics confirmed an important role for these miRNAs in insulin and lipid homeostasis, warranting further work to elucidate their precise role in the exercise-induced improvement in insulin sensitivity. Taken together, our findings suggest that increased miR-155-5p, miR-329-3p and miR-377-3p expression in GSAT is associated with exercise-induced adaptations in lipolysis and inflammation leading to reductions in gynoid fat mass and whole-body improvement in insulin sensitivity. We propose that the exercise-induced expression of miRNAs induce lipolysis, leading to decreased lipogenesis and reduction in gynoid fat mass. These miRNAs may increase insulin sensitivity by activating AKT and IRS1 (Fig. 4).

In contrast to our findings, previous studies on miRNA profiling in adipocytes in response to exercise training have suggested that adipose tissue miRNAs are resistant to exercise-induced changes. A study that investigated miRNA expression in biopsies from GSAT and ASAT found no exercise-induced miRNAs after 6 weeks of endurance training^44^, albeit that this study was conducted in men. Similarly, a study conducted in females, showed no miRNA differences in GSAT and ASAT in response to 16 weeks of high intensity interval training^45^. These contradictory findings may be due to differing exercise intensities and population characteristics such as ethnicity, body distribution, metabolic state, and gender. Notably, in our study, significant miRNA differences were detected in GSAT only, an adipose depot that may be more prominent in black African women than in the Caucasians investigated in the previous studies^46^.

Our study has several strengths. To the best of our knowledge, this is the first study to investigate the effect of an exercise training intervention on miRNA expression in GSAT and ASAT from South African women with obesity and insulin resistance. Exercise-induced miRNAs were identified in GSAT using an unbiased sequencing approach, which identified miRNAs represented in our population. Furthermore, this study investigated the effect of exercise on miRNA expression in adipose tissue, the primary affected organ during obesity development, compared to many other studies which used peripheral blood^13,44^. The participants in this study were advised to maintain their diet, therefore, allowing for the exclusion of dietary influence on miRNA expression. An additional strength of this study is the inclusion of both GSAT and ASAT, thus enabling direct comparison between these depots and their associations with metabolic disease.

Our study also has several limitations to consider. Due to the invasiveness of obtaining adipose tissue biopsies, the samples size is small and may have been underpowered to detect all exercise-induced miRNAs, particularly in ASAT, due to the smaller sample size available for this depot. Therefore, this study is exploratory by nature, and future studies should confirm the findings from this study in larger populations. Furthermore, study participants were South African women with obesity and insulin resistance, therefore we cannot generalize our findings to other populations or males. In addition, miRNAs are epigenetic mechanisms, thus factors such as diet, physical activity, smoking and alcohol consumption^47^ may have confounded our analysis. We did not adjust for these confounders due to the small sample size and risk of overfitting the data. It is also important to acknowledge that adipose tissue consists of adipocytes, mesenchymal stem cells, pre-adipocytes, macrophages, neutrophils, lymphocytes and endothelial cells^48^, therefore we cannot exclude these cells as the source of the exercise-induced miRNAs. Lastly, due to limited tissue availability, we were not able to measure gene expression levels in GSAT, and therefore could not validate the expression of in silico predicted miRNA gene targets.

In conclusion, we showed that a 12-week combined aerobic and resistance exercise training intervention induced adipose-depot specific miRNA expression patterns and improved the metabolic profile of South African women with obesity and insulin resistance. These miRNAs were associated with decreased adiposity and improved cardiorespiratory fitness and metabolic risk, highlighting the important role for depot specific miRNAs in metabolic regulation. Further work in larger samples is required to assess the role of these exercise-induced miRNAs as mediators in adipocyte adaptation to the beneficial effects of exercise training.

## Material and methods

### Participants

Details of the study design have been described previously^16^. This study was approved by the Human Research Ethics Committee at the University of Cape Town (HREC REF:054/2015) and registered in the Pan African Clinical Trial Registry on 21 November 2017 (trial registration: PACTR201711002789113). The study was performed in accordance with the principles of the Declaration of Helsinki (1964, amended 2013) and written, informed consent was obtained from all participants prior to screening and recruitment. The study was conducted over a period of 18 months, between July 2015 and December 2016. Sedentary, obese South African women were recruited through advertisements and selected based on the following inclusion criteria: (1) black, South African women (based on parental Xhosa ancestry) between the ages of 20–35 years; (2) obese (BMI 30–40 kg/m^2^); (3) weight stable (no change in weight more than 5 kg/no change in clothing size over 6 months prior to selection); (4) sedentary (within the last 12 months had not participated in exercise training (more than one session lasting more than 20 min per week); (5) on injectable contraceptive (minimum 2 months; depot medroxyprogesterone acetate 400 mg); (6) no known metabolic/inflammatory disease; (7) no hypertension (≥ 140/90 mm Hg), diabetes (random plasma glucose concentration > 11.1 mmol/L or glycated hemoglobin (HbA1C) > 6.5%); (8) not currently on any medications; (9) non-smokers; (10) not currently pregnant/lactating; (11) no medical problems preventing participation in training; (12) no surgical procedures 6 months prior to study; and (13) human immunodeficiency virus (HIV) negative. A total of 118 women were assessed for eligibility, of whom 73 were excluded due to unwillingness to participate (n = 21), not on contraception (n = 15), BMI < 30 kg/m^2^ (n = 12), BMI > 40 kg/m^2^ (n = 12), HIV positive (n = 2), 20 years old (n = 1), taking medication (n = 1) or being uncontactable (n = 9). A total of 45 women were randomized to the exercise intervention (n = 23) or control group (n = 22). The final sample that was analyzed included 20 exercise and 15 control participants^16^. Three women in the exercise group did not complete the study due to lack of time commitment (n = 2) and becoming pregnant (n = 1) during the study. Seven women in the control group did not complete the study due to loss to follow up (n = 2) and lack of time commitment (n = 5). Based on the availability of samples, only 31 participants (19 exercise and 12 control participants) were included in the current study.

### Intervention

The intervention has been described previously^16,17^. Briefly, women were randomized to receive 12-weeks of supervised aerobic and resistance training at a moderate-vigorous intensity for 40–60 min, four days per week by a trained facilitator (exercise group) or to continue with their usual behaviors (control group). Aerobic exercises included dancing, running, skipping, and stepping at a moderate-vigorous intensity (75–80% peak heart rate). Resistance exercises included upper and lower body exercises using body weight that progressed to the use of equipment (i.e. bands and free weights) at a prescribed intensity of 60–70% peak heart rate. A heart rate monitor (Polar A300, Kempele, Finland) was worn to ensure the prescribed exercise intensity was maintained. The intensity of training was maintained throughout the study by adjusting exercise activity. Both groups were instructed to maintain their usual dietary intake. Following intervention testing, the control participants were given the opportunity to participate in the 12-week supervised exercise program.

### Pre- and post-experimental testing

All participants underwent pre- and post-testing; where cardiorespiratory fitness, body composition, metabolic and biochemical data, alcohol consumption, dietary intake and physical activity were assessed^16,17,27^.

### Cardiorespiratory fitness

Peak oxygen consumption (VO_2_peak) was measured using a walking treadmill-based (C, Quasar LE500CE, HP Cosmos, Nussdorf-Traunstein, Germany) graded exercise test as previously reported^16^. Pulmonary gas exchange was measured by determining O_2_ and CO_2_ concentrations and ventilation to calculate VO_2_ consumption using a metabolic gas analysis system (CPET, Cosmed, Rome, Italy)^16^.

### Body composition

Anthropometric measurements (weight, height, waist and hip circumference), whole body fat composition (including fat mass and fat free soft tissue mass), and regional body fat distribution (gynoid and android fat mass) were assessed using dual-energy-x-ray absorptiometry (DEXA) (Discovery-W, Software version 12.7.3.7; Hologic Inc., Medford, Massachusetts, United States). Visceral adipose tissue and abdominal subcutaneous adipose tissue volumes were analysed using a 3 Tesla whole-body human MRI scanner (MAGNETOM Skyra; Siemens Medical Solutions) using a two-point Dixon method as described previously^17^.

### Frequently sampled intravenous glucose tolerance test

After an overnight fast (10–12 h) blood samples were drawn for the subsequent determination of plasma glucose and serum insulin, triglycerides and TNFα levels. Thereafter, an insulin-modified frequently sampled intravenous glucose tolerance test was used to calculate insulin sensitivity. Briefly, baseline samples were collected at − 5 and − 1 min before a bolus of glucose (50% dextrose; 11.4 g/m^2^ × body surface area) was infused intravenously over 60 s beginning at time 0. At 20 min, human insulin (0.02 U/kg; NovoRapid, Novo Nordisk) was infused over 5 min at a constant rate (HK400 Hawkmed Syringe Pump, Shenzhen Hawk Medical Instrument Co., Shenzhen, China) and samples were collected up to 240 min. Bergman’s minimal model of glucose kinetics was used to calculate the insulin sensitivity index (S_I_)^49^.

### Biochemical analysis

Plasma glucose and serum lipids concentrations were determined using colorimetric assays (Randox, Midrand, Gauteng, South Africa) and serum insulin concentrations were measured using immunochemiluminometric assays (IMMULITE 1000 immunoassay system, Siemens Healthcare, Midrand, Gauteng, South Africa). TNFα concentrations were measured using the Milliplex MAP MAG Human Cytokine kit (Merck, Johannesburg, South Africa) and xMAP technology (Luminex, Austin, Texas, United States) according to the manufacturer’s instruction. Homeostatic model assessment of insulin resistance (HOMA-IR) was calculated using fasting glucose and insulin levels ((glucose (mmol/L) × insulin (pmol/L))/22.5).

### Alcohol consumption

Alcohol consumption was measured as the intake of a standard drink of 10 g of pure alcohol.

### Dietary intake

Pre- and post-experimental period dietary intake was calculated as the average daily intake from a 24-h dietary recall and a 3-day dietary record as previously reported^27^. The 24-h dietary recall was completed with a registered dietitian at pre-testing and every 4 weeks thereafter and the 3-day food records were completed in the same format, including one weekend day.

### Physical activity

Physical activity was objectively measured using accelerometry (ActivPAL, PAL Technologies Ltd, Glasgow, UK) one week before the experimental period (baseline) and again one week before the end of the experimental period (12 weeks). The activPAL was attached to the mid anterior right thigh and worn continuously for 7 days. Data were analyzed using the activPAL software (PAL Technologies, version 7.2.32, Glasgow, UK). Total physical activity was calculated as the sum of light, moderate and vigorous physical activity using reported as minutes/day.

### Adipose tissue biopsies

Adipose tissue biopsies from ASAT and GSAT (2–3 cm^3^) were collected by mini-liposuction after a 4–6 h fast^50^ and at least 48–72 h after the last exercise training session. ASAT samples were obtained from the area directly above the umbilicus, whereas GSAT samples were obtained from the right upper outer quadrant^16^. Samples were washed with saline until no blood was visible, snap frozen in liquid nitrogen and stored at − 80 °C.

### MicroRNA extraction from adipose tissue

MiRNA enriched total RNA was isolated from 100 mg of ASAT and GSAT biopsies using the miRNeasy mini-Kit (Qiagen, Hilden, Germany) according to the manufacturer’s instructions. MiRNA concentrations were measured using the NanoDrop ND-1000 Spectrophotometer (NanoDrop Products, Wilmington, North Carolina, United States), and quality assessed using the Agilent 2100 Bioanalyzer (Agilent Technologies, Santa Clara, California, United States) system and small RNA kits (Agilent Technologies), according to the manufacturer’s instructions.

### MicroRNA sequencing

MiRNAs isolated from 12 GSAT biopsies of six participants, pre- and post-exercise (n = 4) and pre-and post-experiment for controls (n = 2) were sequenced on an Illumina NextGen 500 instrument (Illumina Inc., San Diego, California, United States) using the Illumina TruSeq Rapid SBS preparation protocol (Arraystar Inc., Rockville, Maryland, United States). After quality control, miRNA sequencing libraries were prepared as follows: Total RNA was sequentially ligated to 3′ and 5′ small RNA adapters and complimentary DNA (cDNA) synthesized and amplified using Illumina’s propriety reverse transcription and amplification primers. Amplicons were separated using polyacrylamide gel electrophoresis and amplified fragments corresponding to the molecular size of miRNA fragments with ligated adapters (~ 130 to 150 bp) were excised from the gel for subsequent sequencing. Completed libraries were quantified with an Agilent 2100 Bioanalyzer (Agilent Technologies). The cDNA libraries were diluted to a final concentration of 8 pM and cluster generation was performed on the Illumina cBot using the TruSeq Rapid SR cluster kit, according to manufacturer’s instructions. DNA fragments in the libraries were denatured with 0.1 M NaOH to generate single-stranded DNA molecules, captured on Illumina flow cells, amplified in situ and finally sequenced for 51 cycles on Illumina Nextseq according to the manufacturer’s instruction. Raw sequences were generated as clean reads from Illumina Nextseq by real-time base calling and quality filtering. The clean reads were recorded in FASTQ format, containing the read information, sequences and quality encoding. Subsequently, the 3′ adapter sequence was trimmed from the clean reads and the reads with lengths shorter than 15 nucleotides were discarded. As the 5′-adaptor was also used as the sequencing primer site, the 5′-adaptor sequence is not present in the sequencing reads. The trimmed reads were recorded in FASTA format and were aligned to the human pre-miRNA in miRBase 21 (http://mirbase.org) using Novoalign software (v2.07.11). For miRNA alignment, the maximum mismatch was 1. Reads with counts less than 2 were discarded when calculating miRNA expression. MiRNA expression levels were measured and normalized as transcripts per million of total aligned miRNA reads. The miRNA read counts were used to estimate the expression level of each miRNA. Differentially expressed miRNAs between two groups were analyzed using the two tailed, homoscedastic t-test.

### Quantitative real-time PCR

A total of 10 ng of miRNA-enriched total RNA from GSAT and ASAT was reverse transcribed to cDNA using the TaqMan Advanced MiRNA cDNA Synthesis Kit (Life Technologies, Carlsbad, California, United States), according to the manufacturer’s instructions. Briefly, 5 µl of diluted cDNA (1:10), 1 µl of TaqMan Advanced miRNA Assays (Supplementary Table S2), 10 µl of TaqMan Fast Advanced Master Mix (Life Technologies) and nuclease free water in a total reaction volume of 20 µl were used to quantify miRNA expression using qRT-PCR. Novel miRNA MYN0617 expression was quantified using qRT-PCR with custom-designed TaqMan Assays based on the miRNA sequence obtained by Illumina sequencing (Life Technologies). Briefly, 10 ng of miRNA-enriched total RNA was reverse transcribed using the TaqMan MicroRNA Reverse Transcription kit (Life Technologies). Thereafter, 1.33 µl of cDNA was amplified using 1 µl of Small RNA Assays, 10 µl of Universal PCR Master Mix (no UNG) (Life Technologies), and nuclease free water in a total reaction volume of 20 µl. All qRT-PCR reactions were performed on the QuantStudio 7 Flex Real-Time PCR System using default settings (Life Technologies). The average expression of miR-191 and miR-423 were used as endogenous controls, and miRNA expression levels were calculated using the relative quantification standard curve method.

### In vitro analysis in 3T3-L1 adipocytes

To further investigate the role of differentially expressed miRNAs, we measured their expression in a cell model of obesity^18^. 3T3-L1 adipocytes were exposed to LPS, PA and TNFα, compounds that have been shown to stimulate inflammation and dyslipidemia in 3T3-L1 adipocyte models of metabolic dysregulation. Mouse 3T3-L1 embryonic fibroblasts (American Type Culture Collection, Manassas, Virginia, United States) were cultured in growth medium containing Dulbecco's modified eagle's medium (DMEM, Lonza, Walkersville, Maryland, United States) supplemented with 10% fetal bovine serum (FBS, Thermo Fisher, Waltham, Massachusetts, United States) and cultured at 37 °C in humidified air with 5% CO_2_, and differentiated as previously described^51^. Fully confluent 3T3-L1 pre-adipocytes were seeded at 6 × 10^4^ cells/well in 6-well plates and induced to differentiate by replacing growth media with adipocyte differentiation medium (DMEM supplemented with 10% FBS, 500 µM isobutyl-1-methylxanthine, 1 μg/ml insulin and 1 μM dexamethasone (Sigma-Aldrich, St. Louis, Missouri, United States)) from day 0 to day 3, followed by replacing adipocyte differentiation media with adipocyte maintenance media (DMEM supplemented with 1 µg/ml insulin) and incubating cells for a further 48 h. At day 5, the differentiated adipocytes were cultured in growth medium until they became fully differentiated at day 8 and were subsequently treated with LPS (100 ng/ml, Sigma-Aldrich), PA (750 µM, Sigma-Aldrich) and TNFα (10 ng/ml, Sigma-Aldrich) individually or in combination for 24 h. After treatment, RNA was extracted from 3T3-L1 adipocytes using the AllPrep DNA/RNA/Protein Mini Kit (Qiagen, Hilden, Germany) according to the manufacturer’s instructions. MiRNA expression analysis was conducted using qRT-PCR as previously described, using mouse miRNA assays (Supplementary Table S2).

### Bioinformatics

Bioinformatics was conducted to identify biological pathways affected by the differentially expressed miRNAs. MiRNAs were mapped to pathways based on their mRNA targets using miRNA Pathway Dictionary Database (miRPathDB) 2.0 accessible^52^ at https://mpd.bioinf.uni-sb.de/. miRPathDB identifies pathways from various databases (Gene Ontology, KEGG, miRbase, miRCarta, Reactome and WikiPathways) and gene targets are identified using MiRanda 3.3a, miRTarBase 7 and TargetScan 7.1. Due to the unavailability of sequence information for the novel miRNA in miRPathDB, gene target prediction was conducted using miRDB database, accessible^53^ at http://mirdb.org/. The seed region sequence obtained by sequencing was submitted for gene target prediction. MiRDB implements on the back-end an algorithm, MirTarget, for custom target prediction. The web server collects the miRNA sequence and the selected species from the submission web form. The server thereafter imports all 3’-UTR sequences of the selected species from a precompiled sequence file. It scans for binding sites in the 3’-UTR regions that map and match to the seed region of the miRNA sequence and generates targeting features for MirTarget prediction. The algorithm ranks and attributes a score for each predicted target gene which the server uses to sort the prediction results in descending order for web presentation^53^. The list of target genes was filtered out by considering only genes with a target score above 90%. The output obtained was submitted to the Database for Annotation, Visualisation, and Integrated Discovery (DAVID)^54^ in order to perform disease enrichment analysis focusing only on the genes associated with diabetes. The results obtained from this analysis were further submitted to the KEGG pathway database (https://www.genome.jp/kegg/pathway.html) to identify pathways that respective genes were involved in.

### Statistics

Statistical analysis was performed using STATA version 14.0 (StataCorp, College Station, Texas, United States) and GraphPad Prism® version 8.4.3 (GraphPad Software, San Diego, California, United States). The Shapiro–Wilk test was used to test for normality. Participant data pre- and post-experimental period were compared using the repeated measures mixed model and Bonferroni pairwise analysis. MiRNA data were compared using the Wilcoxon matched pairs signed rank tests. Spearman correlation analyses were conducted to investigate the association between exercise-induced changes in miRNA expression and metabolic characteristics. A P-value ≤ 0.05 was considered statistically significant.

### Ethics approval and consent to participate

This study was approved by the Human Research Ethics Committee at the University of Cape Town (HREC REF:054/2015) and registered in the Pan African Clinical Trial Registry on 21 November 2017 (trial registration: PACTR201711002789113). The study was performed in accordance with the principles of the Declaration of Helsinki (1964, revised 2013). Participants provided written informed consent before screening and participation.

## Supplementary Information

Supplementary Information.

## Data Availability

The datasets generated for this study are available on request to the corresponding author.

## References

[1] Guilherme A, Virbasius JV, Puri V, Czech MP (2008). Adipocyte dysfunctions linking obesity to insulin resistance and type 2 diabetes. Nat. Rev. Mol. Cell Biol..

[2] Pheiffer C (2020). Ethnic and adipose depot specific associations between DNA methylation and metabolic risk. Front. Genet..

[3] Preis SR (2010). Abdominal subcutaneous and visceral adipose tissue and insulin resistance in the Framingham heart study. Obesity (Silver Spring).

[4] Shay CM (2011). Lower extremity fat mass is associated with insulin resistance in overweight and obese individuals: The CARDIA study. Obesity (Silver Spring).

[5] Rantalainen M (2011). MicroRNA expression in abdominal and gluteal adipose tissue is associated with mRNA expression levels and partly genetically driven. PLoS ONE.

[6] Karastergiou K (2013). Distinct developmental signatures of human abdominal and gluteal subcutaneous adipose tissue depots. J. Clin. Endocrinol. Metab..

[7] Ardekani AM, Naeini MM (2010). The role of MicroRNAs in human diseases. Avicenna J. Med. Biotechnol..

[8] Guay C, Roggli E, Nesca V, Jacovetti C, Regazzi R (2011). Diabetes mellitus, a microRNA-related disease?. Transl. Res..

[9] Bartel DP (2004). MicroRNAs: Genomics, biogenesis, mechanism, and function. Cell.

[10] Kim KH, Hartig SM (2022). Contributions of microRNAs to peripheral insulin sensitivity. Endocrinology.

[11] Manoel Alves J (2019). Mapping research in the obesity, adipose tissue, and MicroRNA field: A bibliometric analysis. Cells.

[12] Heyn GS, Corrêa LH, Magalhães KG (2020). The impact of adipose tissue-derived miRNAs in metabolic syndrome, obesity, and cancer. Front. Endocrinol. (Lausanne).

[13] Ehtesham N, Shahrbanian S, Valadiathar M, Mowla SJ (2021). Modulations of obesity-related microRNAs after exercise intervention: A systematic review and bioinformatics analysis. Mol. Biol. Rep..

[14] Tsiloulis, T. & Watt, M. J. Chapter eight—Exercise and the regulation of adipose tissue metabolism. In *Progress in Molecular Biology and Translational Science* (ed. Bouchard, C.) vol. 135 175–201 (Academic Press, 2015). 10.1016/bs.pmbts.2015.06.016 26477915

[15] Mika A, Macaluso F, Barone R, Di Felice V, Sledzinski T (2019). Effect of exercise on fatty acid metabolism and adipokine secretion in adipose tissue. Front. Physiol..

[16] Goedecke JH (2018). An exercise intervention to unravel the mechanisms underlying insulin resistance in a cohort of Black South African Women: Protocol for a randomized controlled trial and baseline characteristics of participants. JMIR Res. Protoc..

[17] Fortuin de Smidt MC (2020). Effect of exercise training on insulin sensitivity, hyperinsulinemia and ectopic fat in black South African women: A randomized controlled trial. Eur. J. Endocrinol..

[18] Jack BU, Mamushi M, Viraragavan A, Dias S, Pheiffer C (2022). Comparing the effects of tumor necrosis factor alpha, lipopolysaccharide and palmitic acid on lipid metabolism and inflammation in murine 3T3-L1 adipocytes. Life Sci..

[19] Hawley JA, Lessard SJ (2008). Exercise training-induced improvements in insulin action. Acta Physiol. (Oxford).

[20] Sakurai T (2013). The effects of exercise training on obesity-induced dysregulated expression of adipokines in white adipose tissue. Int. J. Endocrinol..

[21] Haskell WL (2007). Physical activity and public health: Updated recommendation for adults from the American College of sports medicine and the American Heart Association. Med. Sci. Sports Exercise.

[22] Mota MP (2019). Intervention with a combined physical exercise training to reduce oxidative stress of women over 40 years of age. Exp. Gerontol..

[23] Kelley E (2018). Cardiorespiratory fitness is inversely associated with clustering of metabolic syndrome risk factors: The ball state adult fitness program longitudinal lifestyle study. Mayo Clin. Proc. Innov. Qual. Outcomes.

[24] Bird SR, Hawley JA (2017). Update on the effects of physical activity on insulin sensitivity in humans. BMJ Open Sport Exerc. Med..

[25] Ross R (2020). Waist circumference as a vital sign in clinical practice: A consensus statement from the IAS and ICCR working group on visceral obesity. Nat. Rev. Endocrinol..

[26] Subramanian S, Chait A (2012). Hypertriglyceridemia secondary to obesity and diabetes. Biochimica Biophysica Acta (BBA) Mol. Cell Biol. Lipids.

[27] Clamp LD, Mendham AE, Kroff J, Goedecke JH (2020). Higher baseline fat oxidation promotes gynoid fat mobilization in response to a 12-week exercise intervention in sedentary, obese black South African women. Appl. Physiol. Nutr. Metab..

[28] Rydén M, Arner P (2017). Subcutaneous adipocyte lipolysis contributes to circulating lipid levels. Arterioscler. Thromb. Vasc. Biol..

[29] Vechetti IJJ (2021). Mechanical overload-induced muscle-derived extracellular vesicles promote adipose tissue lipolysis. FASEB J.

[30] Nono Nankam PA (2020). Distinct abdominal and gluteal adipose tissue transcriptome signatures are altered by exercise training in African women with obesity. Sci. Rep..

[31] Nono Nankam PA (2020). Changes in systemic and subcutaneous adipose tissue inflammation and oxidative stress in response to exercise training in obese black African women. J. Physiol..

[32] Velázquez KT (2017). miR155 deficiency aggravates high-fat diet-induced adipose tissue fibrosis in male mice. Physiol Rep.

[33] Gaudet AD (2016). miR-155 deletion in female mice prevents diet-induced obesity. Sci. Rep..

[34] Virtue A (2017). MicroRNA-155 deficiency leads to decreased atherosclerosis, increased white adipose tissue obesity, and non-alcoholic fatty liver disease: A novel mouse model of obesity paradox. J. Biol. Chem..

[35] Lin X (2016). MiR-155 enhances insulin sensitivity by coordinated regulation of multiple genes in mice. PLoS Genet..

[36] Liu S, Yang Y, Wu J (2011). TNFα-induced up-regulation of miR-155 inhibits adipogenesis by down-regulating early adipogenic transcription factors. Biochem. Biophys. Res. Commun..

[37] Mooren FC, Viereck J, Krüger K, Thum T (2014). Circulating micrornas as potential biomarkers of aerobic exercise capacity. Am. J. Physiol. Heart Circ. Physiol..

[38] Zhang Y, Zhang M, Zhong M, Suo Q, Lv K (2013). Expression profiles of miRNAs in polarized macrophages. Int. J. Mol. Med..

[39] Bao F, Slusher AL, Whitehurst M, Huang C-J (2018). Circulating microRNAs are upregulated following acute aerobic exercise in obese individuals. Physiol. Behav..

[40] Ahmadian M (2013). PPARγ signaling and metabolism: The good, the bad and the future. Nat. Med..

[41] Dharap A, Pokrzywa C, Murali S, Kaimal B, Vemuganti R (2015). Mutual induction of transcription factor PPARγ and microRNAs miR-145 and miR-329. J. Neurochem..

[42] Chen L-Y (2018). MicroRNA-377 inhibits atherosclerosis by regulating triglyceride metabolism through the DNA methyltransferase 1 in apolipoprotein E-knockout mice. Circ. J..

[43] Li X (2018). miR-377-3p regulates adipogenic differentiation of human bone marrow mesenchymal stem cells by regulating LIFR. Mol. Cell. Biochem..

[44] Tsiloulis T (2017). Impact of endurance exercise training on adipocyte microRNA expression in overweight men. FASEB J..

[45] Lionett S (2020). Circulating and adipose tissue miRNAs in women with polycystic ovary syndrome and responses to high-intensity interval training. Front. Physiol..

[46] Goedecke JH (2011). Reduced gluteal expression of adipogenic and lipogenic genes in Black South African women is associated with obesity-related insulin resistance. J. Clin. Endocrinol. Metab..

[47] Codocedo JF, Inestrosa NC (2016). Environmental control of microRNAs in the nervous system: Implications in plasticity and behavior. Neurosci. Biobehav. Rev..

[48] Esteve Ràfols M (2014). Adipose tissue: Cell heterogeneity and functional diversity. Endocrinol. Nutr..

[49] Bergman RN, Ider YZ, Bowden CR, Cobelli C (1979). Quantitative estimation of insulin sensitivity. Am. J. Physiol..

[50] Evans J (2011). Depot- and ethnic-specific differences in the relationship between adipose tissue inflammation and insulin sensitivity. Clin. Endocrinol. (Oxf).

[51] Jack BU (2017). A polyphenol-enriched fraction of Cyclopia intermedia decreases lipid content in 3T3-L1 adipocytes and reduces body weight gain of obese db/db mice. S. Afr. J. Bot..

[52] Kehl T (2020). miRPathDB 20: A novel release of the miRNA pathway dictionary database. Nucleic Acids Res..

[53] Chen Y, Wang X (2020). miRDB: An online database for prediction of functional microRNA targets. Nucleic Acids Res..

[54] Jiao X (2012). DAVID-WS: A stateful web service to facilitate gene/protein list analysis. Bioinformatics.

